# Sensor-based gait analyses of the six-minute walk test identify qualitative improvement in gait parameters of people with multiple sclerosis after rehabilitation

**DOI:** 10.1007/s00415-022-10998-z

**Published:** 2022-02-15

**Authors:** Pål Berg-Hansen, Stine Marit Moen, Andreas Austeng, Victor Gonzales, Thomas Dahl Klyve, Henrik Negård, Trine Margrethe Seeberg, Elisabeth Gulowsen Celius, Frédéric Meyer

**Affiliations:** 1 Department of Neurology, Oslo University Hospital, Ullevål, Sognsvannsveien 20, 0372 Oslo, Norway; 2 MS Center Hakadal, Hakadal, Norway; 3 Department of Informatics, University of Oslo, Oslo, Norway; 4 SINTEF Digital, Smart Sensor Systems, Oslo, Norway; 5 Institute of Clinical Medicine, University of Oslo, Oslo, Norway

**Keywords:** Multiple sclerosis, Six-minute walk, Gait analysis, Inertial measurement units, Wearable sensors, Biomechanics

## Abstract

The aim of this work was to determine whether wearable inertial measurement units (IMUs) could detect gait improvements across different disability groups of people with Multiple Sclerosis (pwMS) by the six-minute walk test (6MWT) during a rehabilitation stay in a specialized rehabilitation center. Forty-six pwMS and 20 healthy controls (HC) were included in the study. They performed the 6MWT with two inertial measurement units (IMUs) placed on the feet. Thirty-two of the pwMS were retested at the end of the stay. PwMS were divided in a mild-disability and a moderate-disability group. The 6MWT was divided in six sections of 1 min each for technical analysis, and linear mixed models were used for statistical analyses. The comparison between the two disability groups and HC highlighted significant differences for each gait parameter (all *p* < 0.001). The crossing effect between the test–retest and the two disability groups showed greater improvement for the moderate-disability group. Finally, the gait parameter with the higher effect size, allowing the best differentiation between the disability groups, was the foot flat ratio (*R*^*2*^ = 0.53). Gait analyses from wearable sensors identified different evolutions of gait patterns during the 6MWT in pwMS with different physical disability. The measured effect of a short-time rehabilitation on gait with 6MWT was higher for pwMS with higher degree of disability. Using IMUs in a clinical setting allowed to identify significant changes in inter-stride gait patterns. Wearable sensors and key parameters have the potential as useful clinical tools for focusing on gait in pwMS.

**Supplementary Information:**

The online version contains supplementary material available at 10.1007/s00415-022-10998-z.

## Introduction

Multiple sclerosis (MS) is a chronic disease with onset in young adulthood and characterized by inflammation and neurodegeneration [1]. Walking disability is one of the most common symptoms in people with MS (pwMS) with major impact on physical function and quality of life [2]. The Expanded Disability Status Scale (EDSS) [3] is the most used measure of disability in MS. The scale ranges from 0 (no disability) to 10 (death), heavily depending on ambulation in the higher range and with several limitations [4]. In addition, clinicians use walking tests, both performance-based and patient-reported outcome measures, to assess gait function and the impact of MS on walking. The six-minute walk test (6MWT) [5] is commonly applied to measure endurance walking capacity and has proven to relate better to daily life walking than shorter tests. The 6MWT has been found to detect clinically meaningful improvement after physical rehabilitation [6]. The tests that are used in today´s clinical practice are in general not sensitive enough to detect gait problems and changes in gait function among pwMS with low physical disability, in contrast to the gait measurements and analysis possible in specialized laboratories and research settings [7]. Several studies using automated gait analyses have been published as reviewed by Shanahan et al. [7] and by Brichetto et al. [8]. As an example, it has been shown a greater effect of physical rehabilitation among pwMS with slower gait speed at baseline [9]. Mobility assessment is important in the clinical management of pwMS. Portable technologies with ambulatory monitoring systems and wearable sensors are a rapidly evolving field [10–13]. Sensors can be positioned on different parts of the body depending on the situation. An IMU placed on the waist is probably the most adapted to real-life monitoring [14], while having IMUs placed on the shins or on the foot provide more detailed parameters in a clinical situation [15, 16]. Today inertial measurement units (IMUs) are frequently used in research studies for assessment of movement pattern and stability of persons with gait limitations like elderly that have an increased risk of falling, persons with Parkinson's disease, epilepsy, stroke, and MS [8, 17]. However, wearable unobtrusive sensors may also be useful in clinical settings for example to improve diagnostics, objectively test the effect of interventions and in monitoring disease development. Previous studies have shown that pwMS with moderate or severe walking disability had reduced gait speed, shorter stride length and prolonged swing phase, double limb support time, and stride time, compared to healthy controls (HC) [18]*.* It has also been shown a greater step time variability in pwMS compared to HC [19]. However, there are fewer studies on what characterizes gait in pwMS with mild disability. For example, there has been identified reduced speed, stride length, and double limb support in pwMS with mild disability compared to healthy controls [20, 21]. However, the findings are not consistent, probably due to lack of statistical power and different study populations. A few other studies have utilized body-worn sensors on the 6MWT in pwMS. In a recent review, 5 of 28 studies were on pwMS [22]. Among pwMS with different disability levels, it has been shown that different parameters show clinically meaningful change after rehabilitation [23], and instrumented gait analyses have provided a plethora of metrics for quantifying concurrent factors contributing to gait deterioration and for determining change and responsiveness of interventions [7]. In the present study, we have examined pwMS together with healthy controls (HC) using body-worn IMUs during the 6MWT. The primary aim was to determine whether IMUs could be used to detect improvement in gait parameters across different disability groups during a conventional rehabilitation stay in an MS rehabilitation unit. It was hypothesized that the parameters obtained using wearable sensors would allow for a more sensitive assessment of the pwMS than the standard analysis. We further wanted to investigate the walking impairment in relation to standard walking tests across different disability levels and to explore the potential of IMU-generated parameters as a clinical monitoring tool to detect relevant changes during rehabilitation.

## Methods

### Participants

Several pilots were done in advance to gain information on the number of subjects needed and adjust practical setup. PwMS who were admitted to a rehabilitation stay with focus on any issue that could influence physical function and gait were asked to participate. The participants were allowed to use their own orthoses or other gait aids and had to be able to walk at least 100 m with one cane (EDSS ≤ 6). All that met the inclusion criteria during the study period were asked to participate in the study. Forty-six pwMS aged 26–67 years (mean 50.2) consented and participated (100%). Six of the pwMS had primary progressive MS and 40 had relapsing remitting MS. Thirty-two of the patients were tested twice, at the beginning and end of the rehabilitation stay (mean 15, range 7–22 days). Of these, 26 pwMS were able to walk with no aids, three with one orthosis, and three with canes. The pwMS were divided in a mild-disability group (EDSS < 4) and a moderate-disability group (EDSS ≥ 4). None of the pwMS had changed disease modifying treatment or had any clinical relapses in the last month prior to inclusion. Twelve of the patients were using fampridine which is known to improve walking range in pwMS [24], and of these patients, 10 were retested. One of the participants initiated the drug during the rehabilitation stay and two patients discontinued fampridine. Twenty HC were recruited among employees at the rehabilitation center and were tested once with the same tests protocol as the pwMS. Clinical data for the pwMS, i.e., time of diagnosis and history of immunomodulatory treatment, were retrieved from the medical files.

### Test procedure

All testings were performed in a dedicated test room with a stable temperature between 22 and 23 °C. The hallway was 30 m in length with flat concrete underlay in line with the standard test protocol for the 6MWT [25]. All participants signed the written consent form at inclusion. A neurological exam was performed by a trained neurologist at the beginning of each stay with assessment of EDSS. Prior to the 6MWT, the patients performed four other clinical tests: The time up and go test [26], single leg stand test [27], timed 25-foot walk test [28], and the six spot step test [29]. For the 6MWT, the participants were instructed to walk as far as possible in 6 min along the hallway and turn at each end. They were allowed to take brakes during the test. During the test, they were informed at 3 min that they were halfway through the test, and at 5 min that they had 1 min left.

### Equipment

**Fig. 1:**
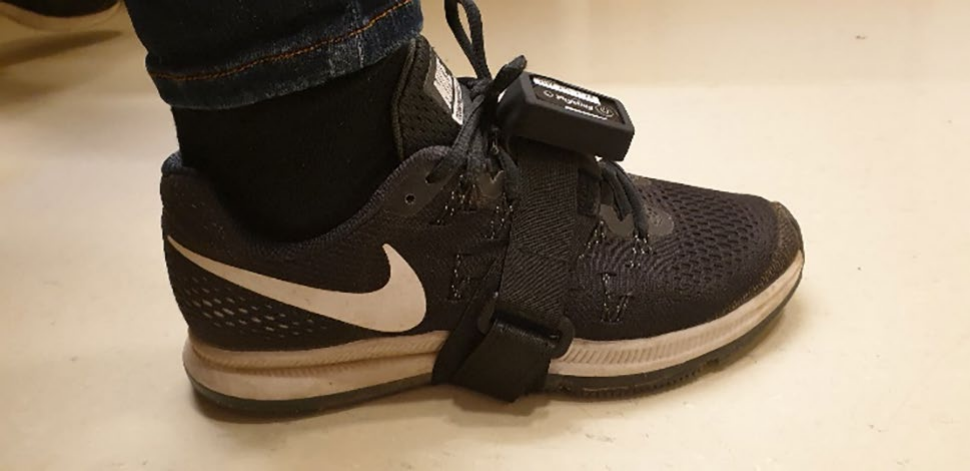
Positioning of the inertial measurement units

Two IMUs (Physiolog 5^®^ from GaitUp SA, Lausanne, Switzerland) containing a 3D accelerometer and a 3D gyroscope were placed centered on the dorsum of each foot with an elastic band (Fig. 1). The sampling rate of the accelerometer and gyroscope was set to 128 Hz with a range of ± 8 g for the accelerometer and ± 1000 °/s for the gyroscope. Fifteen standard gait parameters (i.e., cycle duration, cadence, stance, swing, load ratio, foot flat ratio, push ratio, double support time, stride length, speed, peak swing, foot pitch angle at heal strike, foot pitch angle at toe off, swing width, and path length) were extracted from the IMU data on the feet using a specialized software for gait analysis (GaitUp Lab^®^, GaitUp SA, Lausanne, Switzerland). The description of the parameters can be found in Table 1. The algorithms used to determine these gait parameters have been validated in different publications on healthy [30], elderly [16], and Parkinson [31] population. The sensors were automatically calibrated by the software using an initial static period, as well as the direction of the first steps. The period of each step when the foot is flat on the ground was used to recalibrate the sensor orientation for each cycle [16]. During the analysis, the two steps before and after each turn, as well as the periods of rest, were removed by the software. The movement of each participant was captured with two cameras for back-up. One GoPro Hero 7^®^ camera was mounted on the chest with a GoPro Chesty^®^ strap and directed toward the feet. One GoPro Hero 7^®^ camera was mounted on a tripod directed slightly downwards avoiding the face of the test person to be shown on the film.

**Table 1:**
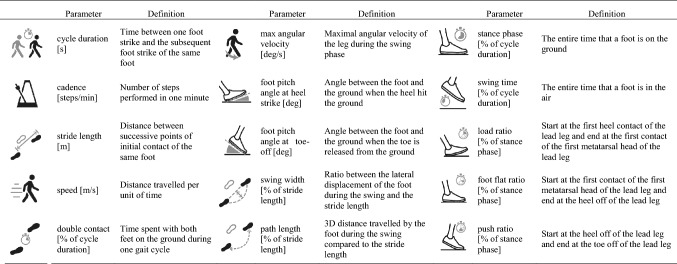
Description of the different parameters determined using the inertial measurement units attached to the feet

### Data transfer and storage

Prior to data analyses, data and videos were transferred for safe storage at the *Service for sensitive data* server. All data handling was unidentified and code key stored separately on an encrypted storage device.

### Statistical analysis

The 6MWT was divided in six sections of 1 min each for technical analysis. To test for normality, a Q–Q plot was used for graphical interpretation and Shapiro–Wilk test was used to test for normality due to small sample sizes. Normally distributed continuous variables were compared using independent samples t test. Mann–Whitney *U* test was used to compare ordinal variables, and Pearson’s Chi-square test was used to compare dichotomous variables. Paired samples t test was used to compare repeated measures. Linear mixed models were used to compare differences in walking parameters (e.g., speed, ground contact time, cadence, loading ratio, and foot flat ratio). The variability was calculated as the difference between the left and the right legs over the whole 6MWT. All the steps included in the global analysis are also included in the variability analysis. Disability group, test–retest, and section were defined as fixed effect and intercept on subject as random effect. Marginal *R*^*2*^ and conditional *R*^*2*^ were presented to assess, respectively, fixed and random effect size. Values over 0.02 were considered small, 0.15 medium, and 0.35 large effect sizes [32]*.* A significance level was set as *p* < 0.05, and a Bonferroni pairwise comparison post hoc test was then applied to identify differences between sections and disability groups. The Bonferroni correction was applied to the *p* value obtained, so the p value is corrected, and the alpha level stays as determined (0.05). The statistical comparisons were obtained using Jamovi Software (Jamovi project 2020, Version 1.2).

## Results

The demographics and clinical data of pwMS and HC are given in Table 2. The total distance walked during the 6MWT was significantly longer for the HC group (mean 694, SD 90 m) compared to the mild-disability group (540, 88 m), who walked longer than the moderate-disability group (421, 99 m) (all *p* < 0.001). After retesting at the end of the rehabilitation stay, the pwMS improved their walking distance by a mean of 15.9 m (*p* < 0.037). The moderate-disability group was able to improve the walking distance by a mean of 24.4 m (SD 38.7, *p* = 0.016) in contrast to 5.1 m (SD 43.4, *p* = 0.67) for the mild-disability group. Test results for the different IMU parameters from the left and right leg averaged for 32 pwMS and 20 HC, were analyzed with the mixed model, and are shown in Table 3. There was a significant difference for all parameters from test to retest, except for load ratio and swing width. For foot pitch angle at heal strike, there was a trend toward significance. Most parameters had medium-to-large effect sizes. The foot flat ratio (percentage of the stance phase with the foot flat) had the highest effect size of 0.53.

**Table 2:** Demographic and clinical data of healthy controls and people with multiple sclerosis

	Healthy controls (*n* = 20)	PwMS All (*n* = 46)	p Value (All pwMS vs healthy controls)	PwMS mild disability (*n* = 20)	PwMS moderate disability (*n* = 26)	*p* Value (mild vs moderate-disability pwMS)
Age in years, mean (SD)	47.7 (12.3)	50.2 (8.3)	0.32	49.4 (10.3)	50.9 (6.5)	0.55
Female/male (% female)	16/4 (80)	29/17 (63)	0.25	15/5 (75)	14/12 (53)	0.14
BMI, mean (SD)	25.4 (3.4)	26.9 (4.4)	0.18	29.1 (3.3)	25.1 (4.4)	0.002
EDSS, median (range)		4.0 (1–6)		2.8 (1–3.5)	4.3 (4–6)	< 0.001
Years since diagnosis, median (range)		10.5 (0–30)		9.0 (0–30)	11 (0–21)	0.77

*PwMS* People with Multiple Sclerosis, *BMI* Body Mass Index, *SD* Standard Deviation, *EDSS* Expanded Disability Status Scale

**Table 3:** Mean spatio-temporal parameters of healthy controls and people with multiple sclerosis

	HC (*n* = 20)	pwMS mild disability (*n* = 14)		pwMS moderate disability (*n* = 18)						Test–retest × section	Test–retest × group	Section × group
	Test	Test	Retest	Test	Retest	r^2^	Test–retest	Section	Group			
Cycle duration (s)	0.87 ± 0.08	0.99 ± 0.10	0.98 ± 0.08	1.09 ± 0.13	1.03 ± 0.12	0.43	< 0.001	0.53	< 0.001	0.93	0.89	0.09
Cadence (steps/min)	140.0 ± 11.8	121.9 ± 11.1	123.2 ± 9.3	111.8 ± 12.6	117.9 ± 12.1	0.45	< 0.001	0.02	< 0.001	0.95	0.63	0.01
Stance phase (% of cycle duration)	58.0 ± 2.4	60.7 ± 2.7	60.7 ± 2.0	62.3 ± 4.6	61.1 ± 3.5	0.39	0.003	0.02	< 0.001	0.58	0.01	0.18
Swing time (% of gait cycle)	42.0 ± 2.4	39.3 ± 2.7	39.3 ± 2.0	37.7 ± 4.6	38.9 ± 3.5	0.39	0.003	0.02	< 0.001	0.58	0.01	0.18
Load ratio (% of stance)	13.1 ± 2.8	10.7 ± 2.4	10.9 ± 2.4	9.0 ± 2.2	9.7 ± 2.3	0.32	0.55	< 0.001	< 0.001	1.0	0.08	< 0.001
Foot flat ratio (% of stance)	41.9 ± 7.5	51.9 ± 6.8	51.9 ± 5.6	59.5 ± 6.9	57.0 ± 5.3	0.53	< 0.001	< 0.001	< 0.001	0.98	0.12	0.03
Push ratio (% of stance)	44.9 ± 6.5	37.4 ± 6.2	37.2 ± 4.8	31.5 ± 6.0	33.4 ± 4.6	0.45	< 0.001	0.60	< 0.001	0.97	0.34	0.80
Double contact time (% of cycle duration)	15.9 ± 4.0	21.1 ± 4.2	21.2 ± 3.7	26.0 ± 11.8	22.9 ± 3.1	0.39	0.001	0.005	< 0.001	0.63	0.008	0.19
Stride length (m)	1.68 ± 0.20	1.53 ± 0.17	1.56 ± 0.13	1.33 ± 0.27	1.40 ± 0.24	0.49	< 0.001	< 0.001	< 0.001	0.85	0.006	0.35
Gait speed (m/s)	1.97 ± 0.29	1.58 ± 0.25	1.62 ± 0.21	1.27 ± 0.33	1.39 ± 0.28	0.52	< 0.001	< 0.001	< 0.001	0.94	0.07	0.03
Max angular velocity (deg/s)	538 ± 52	454 ± 53	463 ± 51	396 ± 82	426 ± 73	0.44	< 0.001	0.38	< 0.001	0.83	0.003	0.50
Foot pitch angle at heal strike (deg)	28.4 ± 5.3	24.6 ± 5.3	26.1 ± 5.4	21.9 ± 6.4	22.9 ± 7.6	0.21	0.07	< 0.001	< 0.001	0.97	0.95	0.43
Foot pitch angle at toe off (deg)	− 84.6 ± 5.9	− 79.3 ± 6.2	− 78.2 ± 5.5	− 67.4 ± 13.9	− 70.8 ± 11.2	0.52	< 0.001	< 0.001	< 0.001	0.78	< 0.001	0.72
Swing width (% of stride length)	0.03 ± 0.02	0.05 ± 0.03	0.05 ± 0.01	0.06 ± 0.03	0.06 ± 0.02	0.02	0.68	0.71	0.77	0.68	< 0.001	0.09
Path length (% of stride length)	105.3 ± 3.4	105.7 ± 6.7	105.0 ± 1.3	109.2 ± 22.1	106.8 ± 3.6	0.19	< 0.001	0.11	0.005	0.23	0.003	0.85

*HC* Healthy control*, PwMS* People with MS

Mean values ± standard deviations are given for the different spatio-temporal parameters of the healthy controls and two pwMS disability groups at test and retest

Statistical analysis of the different parameters, providing the Marginal *r*^*2*^ effect size and the *p* values for the fixed effect and the cross correlations (*x*) between conditions (last six columns)

**Fig. 2:**
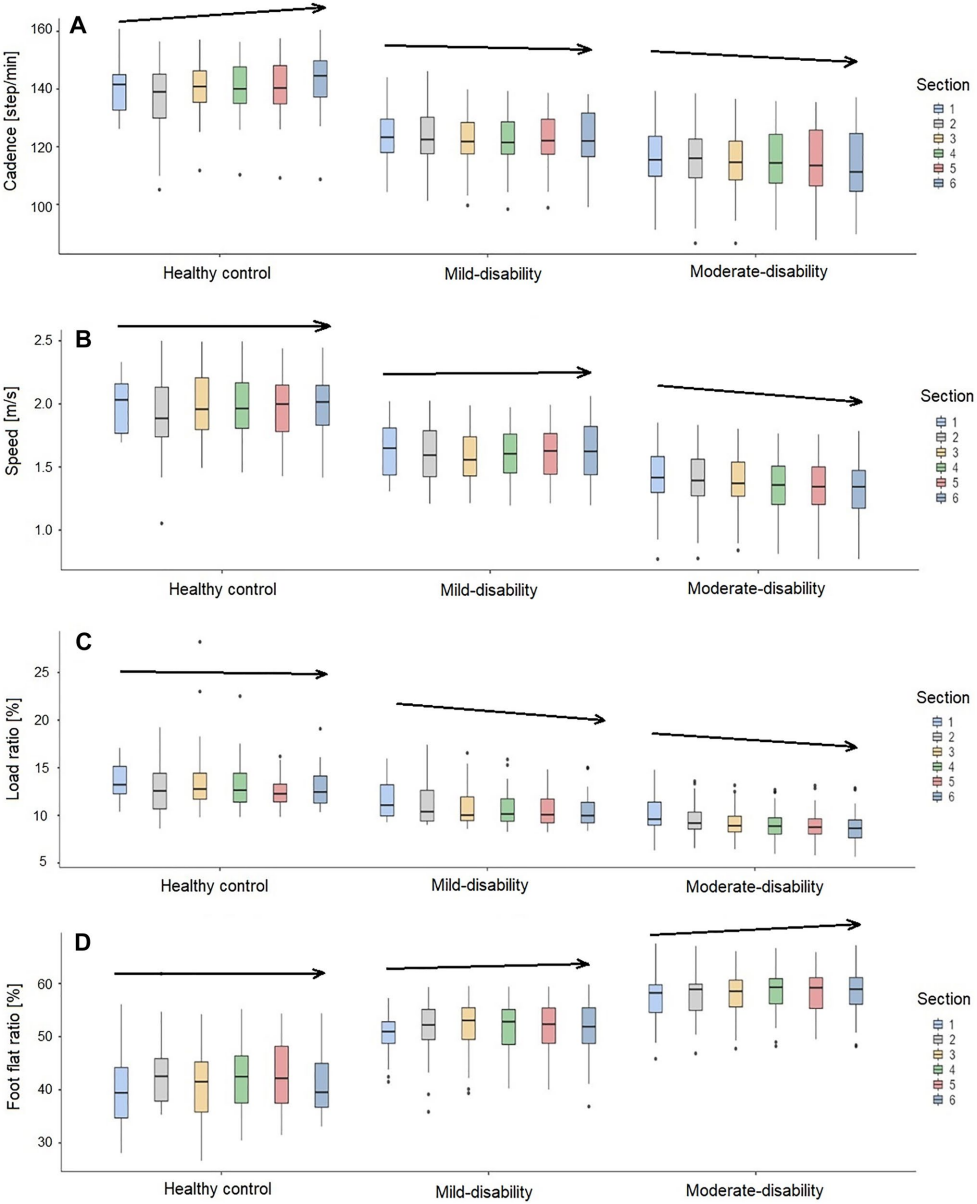
Cross-correlation effect between the groups and the sections of the six-minute walk test. During the six intervals of 1 min each, the cadence (**A**), the speed (**B**), the load ratio (**C**), and the foot flat ratio (**D**) show a different behavior between the healthy controls, the mild-disability, and the moderate-disability groups. Arrows indicate direction of change

There were significant different evolutions of cross-correlation for the parameters cadence, speed, load ratio, and foot flat ratio between the three groups from minute 1 to minute 6 during the test (Fig. 2). The HC group increased cadence, the mild-disability group was stable, and the moderate-disability group decreased cadence during the test. Speed was stable in the HC group and the mildly disabled group but decreased in the moderately disabled group. Load ratio and foot flat ratio were stable in the HC group, whereas for both pwMS groups load ratio decreased, and in accordance with this, foot flat ratio increased during the test. The parameters with non-significant cross-correlation are presented in supplementary material (Figure S1).

**Fig. 3:**
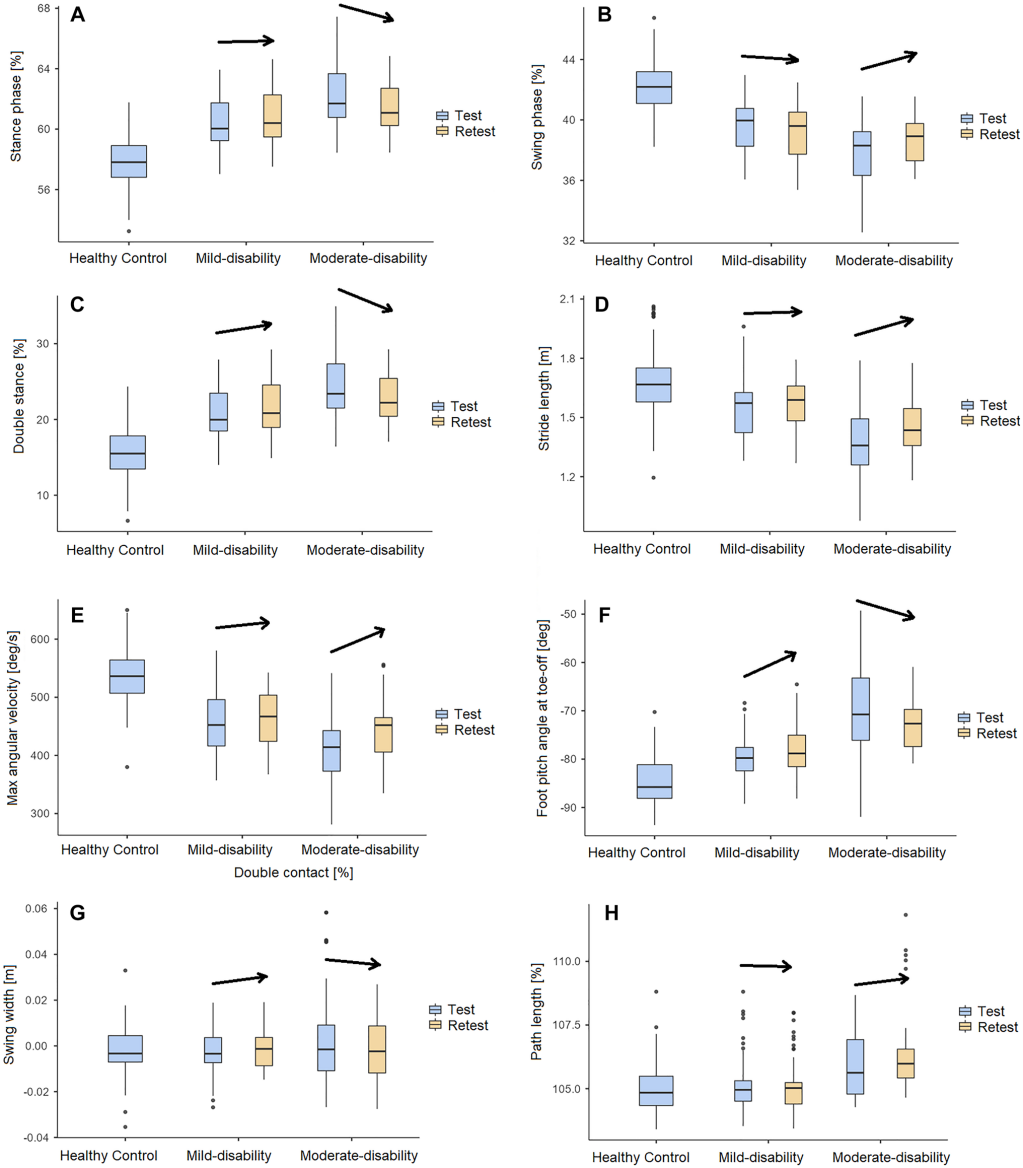
Change in the selected gait parameters stance phase (**A**), swing phase (**B**), double stance (**C**), stride length (**D**),  max angular velocity (**E**), toe-off pitch angle (**F**), swing width (**G**) and path length (**H**) on the six-minute walk test, from baseline test to retest for the mild- and moderate-disability pwMS groups. The HC group was tested only once. Arrows indicate direction of change

Figure 3 illustrates the baseline IMU parameters for all participants, and results at retest for the two disability groups. For the stance phase, swing phase, double stance, stride length, max angular velocity, toe-off pitch angle,  swing width and path length, the moderate-disability group had a better improvement after rehabilitation than the mild-disability group. The parameters with non-significant cross-correlation are presented in supplementary material (Figure S2).

Variability in steps between left and right leg decreased from baseline test to retest, and was significant for the following parameters: stance phase, load ratio, foot flat ratio, push ratio, stride length, gait speed, maximum angular velocity, and path length (Table 4). The correlation coefficients were lower compared to the coefficients obtained for the mean values presented in Table 2, with only small- or medium-effect sizes for all parameters.

**Table 4:** Variability of the spatio-temporal parameters of healthy controls and people with multiple sclerosis

	HC (*n* = 20)	PwMS mild disability (*n* = 14)		PwMS moderate disability (*n* = 18)						Test–retest × section	Test–retest × group	Section × group
	Test	Test	Retest	Test	Retest	r^2^	Test–retest	Section	Group			
Cycle duration (%)	30.2 ± 0.1	0.3 ± 0.1	0.1 ± 0.1	0.8 ± 0.2	0.3 ± 0.1	0.16	0.16	0.012	0.33	0.50	0.87	0.16
Cadence (%)	0.2 ± 0.1	0.3 ± 0.1	0.1 ± 0.1	0.8 ± 0.1	0.2 ± 0.2	0.07	0.41	0.087	0.69	0.93	0.38	0.10
Stance phase (%)	1.7 ± 1.4	2.0 ± 2.0	1.5 ± 1.5	6.4 ± 3.4	4.3 ± 3.0	0.14	0.028	0.54	0.015	0.33	< 0.001	0.006
Swing time (%)	2.4 ± 2.0	3.5 ± 3.7	2.3 ± 2.4	20.3 ± 6.3	7.2 ± 5.5	0.15	0.075	0.64	0.006	0.27	< 0.001	0.008
Load ratio (%)	9.8 ± 7.1	17.7 ± 20.2	21.2 ± 24.7	21.7 ± 21.3	22.9 ± 24.6	0.07	< 0.001	0.75	0.33	0.54	< 0.001	0.36
Foot flat ratio (%)	7.4 ± 5.0	8.5 ± 8.3	10.3 ± 9.5	6.0 ± 4.3	9.3 ± 7.5	0.09	< 0.001	0.541	0.65	0.94	0.018	0.11
Push ratio (%)	6.1 ± 5.4	10.7 ± 8.7	10.2 ± 8.4	16.5 ± 6.4	17.7 ± 5.3	0.10	< 0.001	0.33	0.11	0.94	0.002	0.07
Stride length (%)	3.0 ± 1.5	2.9 ± 1.3	3.0 ± 1.5	21.2 ± 2.1	2.0 ± 2.6	0.05	< 0.001	0.14	0.43	0.69	< 0.001	0.64
Gait speed (%)	3.0 ± 1.5	2.2 ± 3.2	2.7 ± 3.0	6.8 ± 2.1	2.0 ± 2.1	0.05	< 0.001	0.5	0.22	0.38	0.008	0.95
Max angular velocity (%)	3.9 ± 3.0	5.9 ± 7.1	3.8 ± 2.8	15.9 ± 14.3	18.9 ± 18.4	0.19	< 0.001	0.83	< 0.001	0.97	0.006	0.85
Foot pitch angle at heal strike (%)	7.9 ± 4.8	20.3 ± 27.1	16.9 ± 23.0	51.7 ± 19.8	28.6 ± 26.3	0.07	0.25	0.43	0.42	0.44	0.014	0.71
Foot pitch angle at toe off (%)	3.9 ± 2.5	5.5 ± 4.7	4.8 ± 3.3	13.3 ± 13.3	21.9 ± 19.4	0.17	0.99	0.97	0.002	0.29	0.12	0.61
Swing width (%)	160.8 ± 345.4	51.9 ± 106.5	94.6 ± 28.1	38.6 ± 194.4	81.1 ± 144.2	0.10	0.88	0.025	0.005	1.00	0.88	0.004
Path length (%)	0.5 ± 0.4	2.0 ± 1.8	1.1 ± 1.6	6.4 ± 1.3	1.7 ± 1.9	0.09	0.003	0.62	0.057	0.28	0.43	0.85

*HC *Healthy control*, PwMS *People with Multiple Sclerosis

Variability ± Standard Deviations between the left and right leg of the different spatio-temporal parameters for the healthy controls and the two pwMS disability groups at Test–Retest. Statistical analysis of the different parameters, providing the Marginal *r*^*2*^ effect size and the *p* values for the fixed effect and the cross correlations (x) between conditions (last 6 columns).

## Discussion

This is the first study using IMUs in pwMS in relation to standard testing with the 6MWT and as add-on to measure potential effects during a rehabilitation stay. The greater improvement in gait measures was found for the moderate-disability group. The gait parameter with the higher effect size, allowing the best differentiation between the disability groups, was the foot flat ratio (*R*^*2*^ = 0.53). Gait analyses from wearable sensors identified different evolutions of gait patterns during the 6MWT in pwMS with different physical disability. As expected, HC performed better than the mildly disabled pwMS, who again performed better than the moderately disabled pwMS on 6MWT. At the end of the stay, both disability groups walked longer on the 6MWT compared to baseline, but the overall change in the studied heterogeneous group was *lower than the minimum 55 m or more than 20% improvement in walking distance* defined as a minimal clinically important difference by others [33, 34]. However, for the moderate-disability group, it was higher than the clinically meaningful change of 21.6 m estimated for the 6MWT [6]. The immediate measured effect on gait with 6MWT during short-time rehabilitation stay was higher for pwMS with higher degree of disability which is in line with most studies on walking in MS finding the most pronounced efficacy in the more disabled groups [9]. One might speculate that bias toward quantitative data instead of combining qualitative and quantitative data, and measured and perceived (patient- and therapist-reported) data might have influenced the existing data in the field. Moreover, analysis of data from the foot sensors identified significant differences in the gait patterns during the 6 min of the 6MWT between the HC group and the two pwMS groups. The evolution of the cadence, loading ratio, and foot flat ratio combined might be an interesting indicator of the walking ability. The moderate-disability group improved more than the mild-disability group from test to retest. Our findings are partly in line with a study of 58 pwMS with mild (EDSS < 4) and moderate disability (EDSS ≥ 4) [35]. Shema-Shiratzky et al. found that specific gait features deteriorated over the course of the 6MWT, and were related to disability level and other clinical characteristics among pwMS. Subjects with moderate disability walked more poorly in most gait domains compared to the mild-disability group. As in our study, cadence was stable in the mildly disabled group, but decreased in the moderately disabled group during minute 1 and 6. The authors found the same pattern for *sample entropy* but not for other automated gait measures. However, a control group was not included, and there was no retest or intervention. Angelini et al. [36] compared the performance of 6MWT in 57 progressive pwMS and 24 HC by the use of body-worn sensors. Most of the 15 automated gait measures showed good-to-excellent between-session reliability. PwMS had longer step and stride durations/regularity, and had a less stable walk compared to controls. The abnormalities correlated with the level of disability and EDSS scores. The same group recently published an extension of this study [37]. A total of 114 pwMS were compared with 24 HC. Based on the sensor data from 6MWT, they developed a multifactorial model which was able to discriminate clinically relevant differences between pwMS with three disability levels.

A strength of the present study is the use of a standardized test protocol performed in a dedicated test room with stable conditions, without disturbances incorporated in a clinical rehabilitation stay setting. There were also only three, well-trained investigators, and we used a validated, commercially available sensor system with a dedicated software (GaitUp^®^). There were also a relatively high number of pwMS and HC. A limitation of the study was that the HC were not tested twice, due to the restrictions introduced by the COVID-19 regulations. We could thus not rule out a possible learning effect from test to retest. However, the 6MWT is a relatively simple test with a low potential for learning, in contrast to other more complex tests, with reported good-to-excellent between-session reliability [36]. Also, a learning effect only in the more disabled group compared to the less disabled group is unlikely. Our study did not aim to compare different treatment strategies or evaluate therapeutic elements. There were differences in content, duration, and volume of rehabilitation in this *real-world* study with data retrieved during rehabilitation stays of 2–4 weeks. The MS center involved used their standard conventional rehabilitation approach with tailored multidiciplinary content for each participant. As a consequence, the participants were heterogeneous concerning type and number of interventions and goals. The common denominator was that the rehabilitation included a focus on physical rehabilitation related to mobility or walking. Some of the participants worked, e.g., specifically with cognitive behavioral and/or psychological approaches, included in their rehabilitation, so there are many potential factors of interest when searching for the *active ingredients* to improve walking capacity. Despite the heterogeneities, the positive results and trends are striking. If future studies aim to evaluate the specific effects of the interventions on, e.g., walking capacity or gait parameters, core elements of task-specific training, and defined quantitative and qualitative content and goals, adjusted to level of disability, should be included in the results.

There was similar age and BMI distribution in the HC and pwMS; however, there were more females in the HC group, which was adjusted for in the mixed model analysis. The mild-disability group had a higher BMI compared to both HC and the moderate-disability group, which might possibly have interfered with the results. Furthermore, the direction of the finding was in favor of the least disabled group, so that a possible confounder effect would have driven the results into even higher statistical differences. Fampridine may improve gait performance in up to 75% of pwMS [24], and introduction of such treatment during the rehabilitation stay could possibly also have interfered with the results. However, only one patient initiated and two discontinued such treatments during the stay, so fampridine is not considered a major contribution to the results. Three of the pwMS that were tested twice were using orthoses, and three were using canes. Patients using aids like orthoses is part of a real-life clinical patient setting, and we found it relevant to include these as well, though we are aware that this may have affected the gait measures. Unfortunately, the samples sizes were too small to control for this. However, by definition, all the patients that were in need of walking aids were in the moderate-disability group, which had the highest improvement. Since they were using the same device at test and retest, we think that the gait patterns should not have been significantly affected by this. The provided detailed quantitative information on gait pattern compared with the standard speed variable and distance-based walking tests aids tailored feedback and further intervention and monitoring strategies. Thus, the add-on detailed information aids the follow-up and can be investigated as a motivational facilitator, as well. The wearable sensors provide abundant information on gait patterns. Thus, it is crucial to pinpoint a few key spatio-temporal parameters which might be a practical and user-friendly approach. Also, the 6MWT may be burdensome for pwMS with higher degree of disability and be considered too time-consuming in busy clinical settings. Hence, proper adaption of wearable sensors with use of key parameters as a clinical practical application is of great importance, particularly for the potential as a fruitful clinical tool to grasp relevant quantitative aspects.

## Conclusions

The use of wearable sensors as add-on in a conventional rehabilitation setting made it possible to include objective spatio-temporal parameters and qualitative walking assessments. The study identifies significant changes in inter-stride gait patterns which might be relevant qualitative gait changes to assess in a clinical setting. The gait parameter *foot flat ratio*, which allowed better differentiation between the disability groups, might be an interesting parameter in testing and follow-up to pick up change and differentiate concerning walking status. Gait analyses with wearable sensors during clinical testing identified different evolutions of gait patterns during the 6MWT in pwMS with different physical disability and HC. The immediate measured effect on gait with 6MWT during short-time rehabilitation stay was higher for pwMS with higher degree of disability. Standard walking tests do not necessarily measure change in walking capacity. The use of IMUs as add-on also allowed to identify significant changes in inter-stride gait patterns which might be relevant to identify when evaluating walking in pwMS. Thus, wearable sensors with proper adaptation and the use of key parameters have the potential to become useful clinical tools in evaluating and monitoring the disease in different clinical settings.

## Supplementary Information

Below is the link to the electronic supplementary material.

Cross-correlation effect between the groups and the sections of the six-minute walk test for the parameters with non-significant behavior between the healthy controls, the mild-disability, and the moderate-disability groups (TIF 1413 KB)

Gait parameters on the six-minute walk test with non-significant change from baseline test to retest for the mild- and moderate-disability pwMS groups. The HC group was tested only once (TIF 649 KB)
