# Supplementary figures and images for: Sensor-based gait analyses of the six-minute walk test identify qualitative improvement in gait parameters of people with multiple sclerosis after rehabilitation
Source: J Neurol. 2022 Feb 15;269(7):3723–34. doi: 10.1007/s00415-022-10998-z (PMC8853386; doi:10.1007/s00415-022-10998-z)

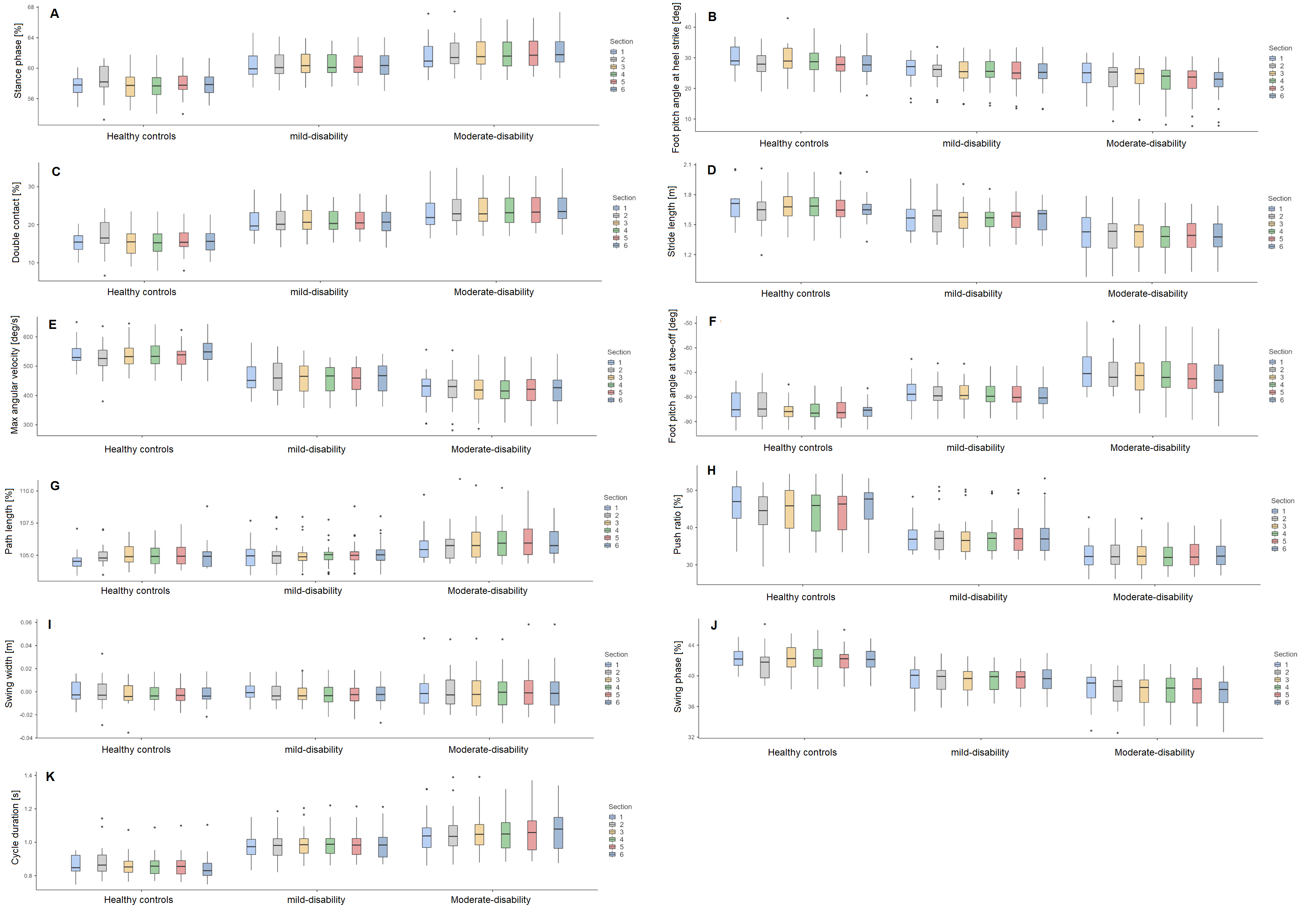

Supplement: Supplementary file 1 — Cross-correlation effect between the groups and the sections of the six-minute walk test for the parameters with non-significant behavior between the healthy controls, the mild-disability, and the moderate-disability groups (TIF 1413 KB) [file 415_2022_10998_MOESM1_ESM.tif]

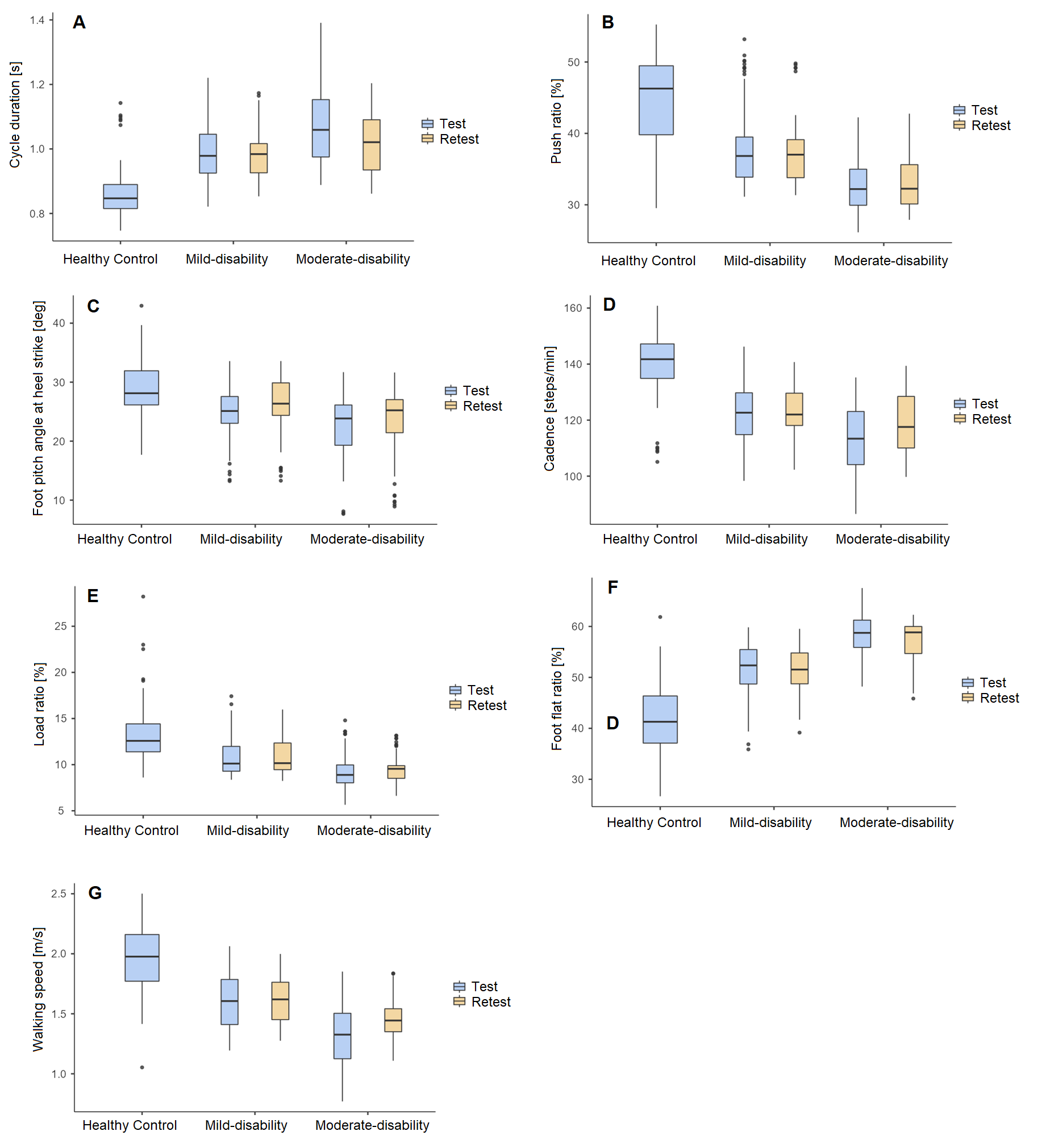

Supplement: Supplementary file 2 — Gait parameters on the six-minute walk test with non-significant change from baseline test to retest for the mild- and moderate-disability pwMS groups. The HC group was tested only once (TIF 649 KB) [file 415_2022_10998_MOESM2_ESM.tif]
